# What can we learn from consumer reports on psychiatric adverse drug reactions with antidepressant medication? Experiences from reports to a consumer association

**DOI:** 10.1186/1472-6904-11-16

**Published:** 2011-10-25

**Authors:** Andreas Vilhelmsson, Tommy Svensson, Anna Meeuwisse, Anders Carlsten

**Affiliations:** 1 Nordic School of Public Health (NHV), Box 121 33, SE-402 42 Gothenburg, Sweden; 2 Department of Behavioural Sciences and Learning, Linkoping University, Linkoping, Sweden; 3 School of Social Work, Lund University, Lund, Sweden; 4 Medical Products Agency, Uppsala, Sweden

## Abstract

**Background:**

According to the World Health Organization (WHO) the cost of adverse drug reactions (ADRs) in the general population is high and under-reporting by health professionals is a well-recognized problem. Another way to increase ADR reporting is to let the consumers themselves report directly to the authorities. In Sweden it is mandatory for prescribers to report serious ADRs to the Medical Products Agency (MPA), but there are no such regulations for consumers. The non-profit and independent organization Consumer Association for Medicines and Health, KILEN has launched the possibility for consumers to report their perceptions and experiences from their use of medicines in order to strengthen consumer rights within the health care sector. This study aimed to analyze these consumer reports.

**Methods:**

All reports submitted from January 2002 to April 2009 to an open web site in Sweden where anyone could report their experience with the use of pharmaceuticals were analyzed with focus on common psychiatric side effects related to antidepressant usage. More than one ADR for a specific drug could be reported.

**Results:**

In total 665 reports were made during the period. 442 reports concerned antidepressant medications and the individual antidepressant reports represented 2392 ADRs and 878 (37%) of these were psychiatric ADRs. 75% of the individual reports concerned serotonin-reuptake inhibitor (SSRI) and the rest serotonin-norepinephrine reuptake inhibitor (SNRI). Women reported more antidepressant psychiatric ADRs (71%) compared to men (24%). More potentially serious psychiatric ADRs were frequently reported to KILEN and withdrawal symptoms during discontinuation were also reported as a common issue.

**Conclusions:**

The present study indicates that consumer reports may contribute with important information regarding more serious psychiatric ADRs following antidepressant treatment. Consumer reporting may be considered a complement to traditional ADR reporting.

## Background

The World Health Organization (WHO) argues that the cost of adverse drug reactions (ADRs) in the general population (in developed countries) is high [1]. Pharmacoeconomic studies from 1997 and 1998 have estimated that ADRs may lead to an additional $1.56 to $4 billion in direct hospital costs per year in the United States [2-4]. These estimations are however uncertain and in most countries the extent of this expenditure has not been measured. The reporting of potential ADRs by healthcare professionals is supported by WHO and their Drug Monitoring Programme [5], and under-reporting by health professionals is a well-recognized problem by the WHO [6]. Another way to increase ADR reporting could be to let the consumers themselves report directly to the authorities.

One important step towards consumer reporting of ADRs was recently taken by the European Parliament, who in September 2010 voted in favor for a new pharmacovigilance legislation to ensure greater patient safety and to improve public health [7,8]. This was later cleared by the European Council in December 2010 [9]. The new legislation came into force on 1 January 2011 but will not apply until July 2012 [7,9]. Member States will then have to adopt these changes in order to harmonize national adverse event systems, and one important change to the current law foresees the inclusion of direct patient reporting (DPR) of adverse events [10]. Some mean that this will mark the beginning of a new chapter in drug safety [11]. The WHO acknowledges that it is not always easy to recognize ADRs (which may act through the same physiological and pathological pathways as different diseases) and proposes a step-wise procedure to assessing possible drug-related ADRs [6]. Therefore, the organization proclaims consumer reporting to be of great importance in order to safeguard a pharmacovigilance that will help each patient to receive optimum therapy, and on a population basis will lead to ensure the acceptance and effectiveness of public health programmes [12].

Consumers in both the Netherlands and Denmark have had the possibility to report ADRs to their authorities since 2003. Different studies have shown that ADRs reported by patients has the potential to increase knowledge about the possible harm of medicines [10]. A Danish study of reports to the Danish Medicines Agency (DKMA) showed for instance that patients are more likely to report ADRs from the nervous and psychiatric system than are health professionals [13]. A Dutch study indicated that patients seem to report different experiences compared to healthcare professionals regarding ADRs from antidepressants [14], and that patients now take great interest in their drug use and often search for more information about their own medication and often focus on ADRs [15]. Withdrawal symptoms are according to an English study described in a clearer way by consumer reports compared to how it was done by the National drug regulatory agencies [16]. However, very few studies have compared 'real life' reports made by patients and health professionals about antidepressants [10,14,16,17]. Previous research in the Netherlands and in Denmark has also suggested that consumer experiences should be included in the evaluation of antidepressant treatment in clinical practice [14], and in systematic drug surveillance systems [13].

In the Nordic countries sales of antidepressants has increased up to four fold since the middle of the nineties [1], and the sales has now stabilized [18] (Table 1). In Sweden in 2010 approximately 8.1% of the population did purchase an antidepressant drug and more than five million prescriptions of antidepressants were dispensed to almost 760 000 patients (66% were women) [19]. In 2009 the estimated sales for antidepressants in Sweden were almost 70 million Euros [20]. Increased use may be followed by a higher incidence of adverse drug reactions (ADRs) and pharmacovigilance is therefore considered important aiming to make the best use of medicines for the treatment or prevention of disease [12]. In a societal perspective increased knowledge of this kind is of great importance. In Sweden drug-related problem may account for as much as 12% of hospital admissions [21] and the medical burden of fatal ADRs is estimated to occur in 3% of all deaths [22]. Antidepressants drugs are commonly implicated in FADRs [23].

**Table 1:** Sales of antidepressants (N06A) in the Nordic Countries during 1995-2008 in DDD*/1000 inhabitants per day [1,35]

	Denmark	Finland	Iceland	Norway	Sweden
1995	18.3	20.3	33.0	22.5	27.8
2000	34.7	35.5	70.5	41.0	48.8
2004	55.2	49.9	91.9	52.4	64.6
2005	59.9	52.1	94.8	51.8	66.1
2006	64.7	55.5	92.6	52.7	69.7
2007	69.9	61.1	95.4	54.8	72.1
2008	73.4	63.9	94.7	55.1	73.7

* Defined Daily Doses according to WHO classification

In Sweden it is mandatory for prescribers to report potential serious ADRs to the Medical Products Agency (MPA) [24]. There are however no such regulations for consumers. Despite the possibility for consumers to report potential ADRs to the MPA the number of incoming reports is quite few. The information about how patients perceive their treatment with antidepressants and their perception of ADRs is scarce. The non-profit and independent organization Consumer Association for Medicines and Health, KILEN has launched the possibility for consumers to report their perceptions and experiences from their use of medicines in order to strengthen consumer rights within the health care sector. KILEN established a consumer database already in 1997 to collect consumer reports mainly focusing on benzodiazepines and antidepressants. KILEN was created in 1992 but their co-workers had already a long history of working with pharmaceutical drug dependency when it in the 1960s became clear that the new benzodiazepines were causing dependency and harm. Since 2002 it has also been possible to report experiences with medicines to KILEN through a web based report form (http://www.kilen.org). These reports have not yet been scrutinized and analyzed. Hence, this study aimed to analyze these consumer reports.

## Methods

All reports submitted from January 2002 to April 2009 to KILEN's internet-based reporting system in Sweden were analyzed. Main focus in the study was common ADRs related to antidepressant usage. According to WHO an ADR is defined as a response to a medicine which is noxious and unintended, and which occurs at doses normally used in man whilst an adverse event or experience is defined as any untoward medical occurrence that may present during treatment with a medicine but which does not necessarily have a causal relationship with this treatment [6].

A report in the KILEN material was defined as one individual's reported experience with a drug and an ADR was equal to one single reported effect connected to a specific drug. The report form items included user information (age, sex, location and condition of health), the story about the treatment (medical history, drugs, doses and reactions). It was also possible to give a longer description of the experience as free text. In this study we chose to analyze age, sex, drug reported and ADRs. More than ADR related to the same drug could be submitted. The reported ADRs to KILEN were compiled and coded in a similar way to those listed in the Swedish Physicians' Desk Reference, FASS. FASS is building on the Summary of Product Characteristics (SPC) from the pharmaceutical companies. KILEN personnel using the database software FileMaker did this coding.

The drugs in reports were coded according to therapeutic groups [Anatomical Therapeutic Chemical (ATC) system] [25] and types of reported ADRs (system organ classes) [26]. The ATC Classification with Defined Daily Doses (ATC/DDD) system classifies therapeutic drugs and the purpose of the system is to serve as a tool for drug utilization research in order to improve quality of drug use [25]. In the ATC classification system, the drugs are divided into different groups according to the organ or system on which they act and their chemical, pharmacological and therapeutic properties [25]. This system is also valid for the Swedish Physicians' Desk Reference, FASS. The ADRs in FASS are classified according to MedDRA system organ class where reactions are reported corresponding to their frequency (Very common = >10%, Common = 1-10%, Less common = 0.1-1%, Rare = 0.01-0.1%, Very rare = <0.01%, Unknown frequency). Data submitted to KILEN are not handled to the regulatory authorities like the MPA. The project was approved by the ethics board in Gothenburg, Sweden (No. 319-10).

## Results

In total 665 individuals submitted reports on ADRs to a specific drug and 469 of these concerned antidepressants. Fifteen different antidepressant drugs were reported but for eight of these antidepressants too little information (≤10 individual reports) was available. The 442 individual antidepressant reports included in the study represented 2392 ADRs and of these were 878 psychiatric ADRs (37%) (Table 2). 75% of the individual reports concerned serotonin-reuptake inhibitor (SSRI) and 25% a serotonin-norepinephrine reuptake inhibitor (SNRI). The most reported antidepressants to KILEN were: Sertraline (26%), Citalopram (24%), Venlafaxine (18%), Paroxetine (13%), Mirtazapine (8%) Fluoxetine (6%), and Escitalopram (5%). Sertraline and Citalopram were the most common antidepressants according to both reports (116 and 107), total ADRs (626 and 570) and psychiatric ADRs (226 and 226) (Table 2). Women were responsible for 323 of the submitted reports compared to men with 98 reports. 21 individuals did not report their gender and eight individuals did not submit their age. Of the psychiatric ADRs were women responsible for 622 (70.8%) and men 208 (23.7%), whilst 5.5% did not report their gender (Table 3). The distribution of ADRs per report was quite even between women (5.4) and men (5.2). A majority (34.5%) of the antidepressant psychiatric ADRs were reported by consumers within the age group 30-39 years of age (women 26.8% and men 6%) (Table 3). Also age groups 15-29 years of age (23.6%) and 40-49 years of age (22.1%) were common reporting groups. Several reports to KILEN included withdrawal symptoms, where one fourth to one third of psychiatric ADRs were reported during discontinuation (Table 4). Women were responsible for a majority of the reports within the different antidepressants (65.3-82.7%) compared to men (12.2-28.9%) (Table 5). Only Mirtazapine was more evenly reported (52.1 compared to 43.5%).

**Table 2:** Reports and ADRs of antidepressant medication to an open web site according to the system organ class of psychiatric system^1^.

Antidepressant ATC code N06A	Reports (N) Total = 442	ADRs (N) Total = 2392	Psychiatric ADRs (N) Total = 878	ADRs/report	Most common psychiatric ADR (%)
Sertraline^a^	116	626	226	5.4	Anxiety 5.9
N06AB06					Sensation of unreality 4.0
					Insomnia 3.0
					Uneasiness/nervousness 2.6
					Irritability, aggressiveness 2.2
					Suicidal behavior 1.9

Citalopram^a^	107	570	226	5.3	Anxiety 7.9
N06AB04					Insomnia 3.7
					Sensation of unreality 2.8
					Suicidal behavior 2.5
					Uneasiness/nervousness 2.5
					Depression 2.1
					Irritability, aggressiveness 2.1

Venlafaxine^b^	78	505	171	6.5	Anxiety 4.2
N06AX16					Suicidal behavior 3.2
					Uneasiness/nervousness 2.8
					Sensation of unreality 2.8
					Insomnia 2.4

Paroxetine^a^	58	327	121	5.6	Anxiety 5.2
N06AB05					Irritability, aggressiveness 3.4
					Suicidal behavior 3.1
					Insomnia 2.3
					Depression 2.1

Mirtazapine^b^	34	131	46	3.9	Anxiety 6.9
N06AX11					Insomnia 6.1
					Irritability, aggressiveness 3.1
					Suicidal behavior 2.3
					Uneasiness/nervousness 2.3

Fluoxetine^a^	28	120	39	4.3	Anxiety 5.0
N06AB03					Irritability, aggressiveness 2.5
					Suicidal behavior 2.5
					Insomnia 2.5

Escitalopram^a^	21	113	49	5.4	Anxiety 7.1
N06AB10					Sensation of unreality 6.2
					Insomnia 5.3
					Depression 3.5
					Irritability, aggressiveness 3.5
					Suicidal behavior 2.7
					Uneasiness/nervousness 2.7

^1^According to ATC classification system, the drugs are divided into different groups according to the organ or system on which they act and their chemical, pharmacological and therapeutic properties.

^a^Selective serotonin reuptake inhibitor (SSRI)

^b^Serotonin-norepinephrine reuptake inhibitor (SNRI)

**Table 3:** Consumer reported psychiatric ADRs (N = 878) to KILEN according to age and gender (N) and (%)

Gender	Age group								
	**15-29**	**30-39**	**40-49**	**50-59**	**60-69**	**70-79**	**80-89**	**No age**	**Total**

Female	150 (17.1)	235 (26.8)	151 (17.2)	42 (4.8)	17 (1.9)	10 (1.1)	-	17 (1.9)	622 (70.8)
Male	46 (5.2)	53 (6.0)	64 (7.3)	30 (3.4)	10 (1.1)	-	3 (0.3)	2 (0.2)	208 (23.7)
Not given	9 (1.0)	16 (1.8)	14 (1.6)	-	-	-	-	9 (1.0)	48 (5.5)
Total	550 (23.6)	804 (34.5)	515 (22.1)	242 (10.4)	85 (3.6)	23 (1.0)		44 (1.9)	878

**Table 4:** Reported antidepressant psychiatric ADRs to KILEN during different stages of treatment

Type of reported psychiatric adverse drug reaction and frequency (N)	During treatment (%)	During discontinuation treatment (%)	After treatment (%)
Anxiety (139)	40	34	26
Sensation of unreality (57)	54	25	21
Insomnia (72)	54	28	18
Uneasiness/nervousness (50)	50	30	20
Irritability/aggressiveness (45)	49	33	18
Suicidal behaviour (59)	68	19	13
Depression (20)	25	45	30

**Table 5:** Internet reported antidepressant psychiatric ADRs to KILEN according to age and gender (%)

	Sertraline	Citalopram	Venlafaxine	Paroxetine	Mirtazapine	Fluoxetine	Escitalopram
Gender							
Female	67.3	82.7	68.4	67.8	52.1	71.8	65.3
Male	27.8	16.4	21.6	28.9	43.5	25.6	12.2

Age group							
15-29	26.5	12.4	20.5	33.1	37.0	30.8	26.5
Female	16.8	9.7	15.8	24.0	37.0	30.8	10.2
Male	7.1	2.6	4.1	9.1	-	-	12.2
30-39	45.5	39.8	27.5	30.6	14.0	17.9	28.6
Female	27.4	36.7	27.5	23.1	-	2.6	28.6
Male	13.3	2.2	-	5.0	14.0	15.4	-
40-49	12.8	27.9	38.6	28.1	4.6	38.5	40.8
Female	11.8	21.2	19.3	13.2	2.3	35.9	24.5
Male	0.9	6.6	15.8	14.9	2.3	2.6	-
50-59	7.5	12.4	5.5	3.3	26.4	2.6	2.0
Female	2.6	8.4	3.6	3.3	11.1	2.6	2.0
Male	4.9	4.0	1.9	-	15.3	-	-
60-69	4.9	3.5	-	1.6	14.9	-	-
Female	4.0	2.6	-	1.6	-	-	-
Male	0.9	0.9	-	-	14.9	-	-
70-79	2.0	-	-	-	-	-	-
Female	2.0	-	-	-	-	-	-
Male	-	-	-	-	-	-	-
80-89	-	-	-	-	-	7.7	-
Female	-	-	-	-	-	-	-
Male	-	-	-	-	-	7.7	
No age given	0.8	4.0	7.9	3.9	3.1	2.5	2.1

The most frequently reported psychiatric ADRs to KILEN were anxiety, a sensation of unreality, insomnia, uneasiness/nervousness, irritability, aggressiveness, suicidal behavior, and depression (Table 2). The most common ADR was anxiety (4.2-7.9%). Insomnia was reported for all antidepressants to KILEN (2.3-6.1%). The ADR uneasiness/nervousness was reported for five antidepressants (2.3-2.8%). Experiencing a sensation of unreality was a common ADR in four analyzed antidepressants (2.8-6.2%). Depression was a reported psychiatric ADR in three antidepressants (2.1-3.5%). Irritability, aggressiveness was a reported psychiatric ADR for six antidepressants (2.1-3.5%). Suicidal behavior was a reported psychiatric ADR for all antidepressants in the KILEN material (1.9-3.2).

## Discussion

The KILEN material showed reports of potentially serious psychiatric ADRs. Some psychiatric ADRs were more reported with certain antidepressants but anxiety, insomnia and suicidal behavior were reported for all drugs. But do these consumer reports differ according to information found in the Summary of Product Characteristics (SPC)? If we compare reports to KILEN between the years 2002-2009 with FASS 2004 [27] and FASS 2009 [28] we take in consideration that it often takes years before new ADRs are published in FASS. FASS is the most used tool for health care professionals in Sweden to use when prescribing drugs and therefore of interest in a comparison with consumer reports. ADR information in FASS is mainly based on information from the pharmaceutical companies and a somewhat good correspondence to the consumers' reports is expected. However, the consumer reports gave another perspective of experiences with antidepressants. The consumer reports to KILEN contained more potentially serious psychiatric ADRs that are not always listed in FASS, especially experiencing a sensation of unreality, irritability, aggressiveness, suicidal thoughts, and depression. Anxiety was the most reported psychiatric ADR to KILEN for all antidepressants but for some substances anxiety is not listed at all in one version of FASS, but listed as common in the other.

This result goes well in accordance with previous research that consumer/patient reporting does add value to professional reports of ADRs by identifying possible new reactions [13,14,29,30]. For instance was a sensation of unreality an important psychiatric ADR among the consumer reports to KILEN, but is not listed at all as an ADR in FASS. Withdrawal symptoms in connection with discontinuation of antidepressants medication was reported to KILEN but is not always mentioned in FASS, and when it is mentioned it is generally regarded as rare [27,28]. This is worth considering since a study by Tint and colleagues (2008) showed that withdrawal symptoms of antidepressants in depressed patients could be associated with worsening depression symptoms and increasing suicidal ideation [31].

Consumer reporting may be one way of picking up harms that are missed in clinical trials, where for instance the KILEN material introduces a common self reported harm in experiencing a sensation of unreality. The new legislation in the EU-countries to stimulate a systematic consumer reporting can therefore be an important step to take, and hopefully will also the newly established consumer reporting system to the Swedish Medical Product Agency lead to a safer prescription culture. Since the start in 2008 and up until November 2010 the agency has received over 4000 consumer reports, according to the MPA. There is however a major uneven distribution due to the vaccination campaign during the A(H1N1) pandemic in 2009. Research has also shown that educating general practitioners (GPs) to focus on ADRs improved the ADR reporting [32]. This is particularly serious since only five percent of doctors are estimated to participate in any pharmacovigilance system [33]. Educating physicians more in pharmacology or an active involvement of pharmacists when prescribing medication may therefore be one way to minimize ADRs and thereby increase safety. Maybe increased consumer reporting can lead to an increase in ADR reporting from health professionals. Distribution of start packages of antidepressant medication can also be one important aspect in a safer prescription culture and an important step in minimizing ADRs through better follow-up. However, as Danish research suggests, can consumer ADR report might act as whistleblowers of new and previously undetected ADRs, but if the quality of the reports is questionable they may bring too much noise rather than valuable information to the pharmacovigilance systems [34].

Gender is also an important issue to highlight since the sales of antidepressants are almost twice as high among women compared to men in all age groups [35]. Women reported ADRs to KILEN in a much higher degree, between three to four times more often than men, and sometimes more within certain age groups. Especially women 30-39 years of age was a large frequently reporting group, but also younger women (15-29 years of age) was a common group. This may be an effect of that maybe women to a higher degree turn to non-profit organizations for help. It can also be an effect of women tending to have a higher risk of ADRs than men, which increases with age and increased numbers of drugs prescribed [36]. Citalopram was in particular a commonly reported antidepressant medication by women answering for almost 83% of the psychiatric ADRs for this drug. Both suicidal behavior and depression, which are more frequently associated with women, were commonly reported psychiatric ADRs for Citalopram in the KILEN material. There is an almost two-fold higher occurrence of lifetime prevalence of major depressive disorder and anxiety disorders in females than in males [37], and older women with a previous history of treatment by a psychiatrist may have an increased risk of becoming long-term users of antidepressants [38]. Since depression was a highly reported psychiatric ADR during discontinuation in the KILEN material it may be of importance to include consumer reports when prescribing the drug of choice for instance depression.

However, this study does have several limitations. There is for instance the question of potential problems with polypharmacy, with an unknown interaction between psychotropic drugs, for instance different antidepressants and anxiolytics. As indicated by a Swedish study the prevalence of polypharmacy, as well as the mean number of dispensed drugs per individual, increased for instance year-by-year in Sweden 2005-2008 [39]. Hence we cannot know for sure if the reported consumer reports do contain specific psychiatric ADRs for one antidepressant drug alone or if it is a combined effect due to other drugs. Some of the antidepressants have quite few reports (Mirtazapine, Fluoxetine and Escitalopram) and therefore it is not possible to draw any conclusions for each medication. It is a strength with the KILEN material that consumers are asked to fill in the report form concerning other medications as well, but it is still difficult to know if the reported ADR is a result of a specific medication or the combination of a number of medications. This is however not unique for KILEN but also valid for the report form from the MPA. Nevertheless, consumer reporting may make an important contribution in gaining information about unknown drug interactions. The KILEN data was based on spontaneous consumer reports and thereby a selected material, which may enhance a negative view of antidepressant medication. Despite these limitations the study is still of value since the material gives us unique information of consumer reporting in Sweden.

## Conclusions

The present study indicates that consumer reporting may contribute with important information. Consumer reporting may be considered a complement to traditional ADR reporting.

## Competing interests

The authors declare that they have no competing interests.

## Authors' contributions

AV and AC were responsible for study concept and design. AV acquired the data. AV and AC interpreted the data. AV drafted the manuscript and all authors contributed with critical revisions of the manuscript. All authors read and approved the final manuscript.

## Pre-publication history

The pre-publication history for this paper can be accessed here:

http://www.biomedcentral.com/1472-6904/11/16/prepub
